# Hypoxia promotes the proliferation of mouse liver sinusoidal endothelial cells: miRNA–mRNA expression analysis

**DOI:** 10.1080/21655979.2021.1988371

**Published:** 2021-10-21

**Authors:** Zhe Qing, Hanfei Huang, Qun Luo, Jie Lin, Shikun Yang, Tao Liu, Zhong Zeng, Tingfeng Ming

**Affiliations:** a Organ Transplantation Center, the First Affiliated Hospital of Kunming Medical University, Kunming, Yunnan Province, China; b Department of Pediatrics, The First Affiliated Hospital of Kunming Medical University, Kunming, Yunnan, China

**Keywords:** Liver sinusoidal endothelial cells, microrna, liver regeneration, hypoxia, bioinformatics

## Abstract

During the initial stage of liver regeneration (LR), hepatocytes and liver sinusoidal endothelial cells (LSECs) initiate regeneration in a hypoxic environment. However, the role of LSECs in liver regeneration in hypoxic environments and their specific molecular mechanism is unknown. Therefore, this study aimed to explore the miRNA–mRNA network that regulates the proliferation of LSECs during hypoxia. In this study, first, we found that the proliferation ability of primary LSECs treated with hypoxia was enhanced compared with the control group, and then whole transcriptome sequencing was performed to screen 1837 differentially expressed (DE) genes and 17 DE miRNAs. Subsequently, the bioinformatics method was used to predict the target genes of miRNAs, and 309 pairs of interacting miRNA–mRNA pairs were obtained. Furthermore, the miRNA-gene action network was established using the negative interacting miRNA–mRNA pairs. The selected mRNAs were analyzed by Gene Ontology (GO) enrichment analysis and Kyoto Encyclopedia of Genes and Genomes (KEGG) analysis, and biological processes (BP) and signal pathways related to LSEC proliferation that were significantly enriched in GO-BP and KEGG were selected. Finally, 22 DE genes and 17 DE miRNAs were screened and the network was created. We also successfully verified the significant changes in the top six genes and miRNAs using qRT-PCR, and the results were consistent with the sequencing results. This study proposed that a specific miRNA–mRNA network is associated with hypoxia-induced proliferation of LSECs, which will assist in elucidating the potential mechanisms involved in hypoxia-promoting liver regeneration during LR.

## 1. Introduction:

After acute injury or liver resection, multiple types of cells, including hepatocytes and non-parenchymal cells, participate in liver regeneration (LR) under precise regulations to quickly restore liver function, and this process is very complex [1–3]. Liver sinusoidal endothelial cells (LSECs) are the largest type of liver non-parenchymal cells after hepatocytes [4]. During regeneration, LSECs provide nutrients and oxygen for hepatocyte proliferation through autocrine and paracrine secretion of various cytokines and the formation of new blood vessels [5–8]. LSECs play an important role in LR by maintaining the relative homeostasis of the sinusoidal environment and creating an environment conducive to regeneration [9,10].

Studies have confirmed that LR occurs in a hypoxic environment, and thus, hypoxia can promote LR [11–14]. At the same time, recent literature has also reported that hypoxia can promote the proliferation of LSECs and maintain the integrity of hepatic sinusoid structure, thus promoting hepatocyte proliferation [13]. However, the specific molecular mechanism of LSEC proliferation and the promotion of LR under hypoxia has not been elucidated. Furthermore, it has not been reported whether this is related to the differential expression and interaction of messenger RNAs (mRNA) and micro RNAs (miRNAs). Therefore, we speculated that there are differential changes in miRNA and mRNA in LSECs during hypoxia, which may participate in the regulation of LSEC proliferation or other biological pathways, and may eventually affect LR.

miRNAs are a small group of non-coding RNAs that bind to specific target mRNAs and regulate gene expression; it has been confirmed to be involved in the regulation of LR [15–17]. However, there are no reports on the changes in miRNA and mRNA levels in LSECs during hypoxia and their possible involvement in the regulation of LR. Based on previous reports on the initial stages of LR, in this study, the process of mouse hepatocyte regeneration was carried out in a hypoxic environment. We also analyzed whether the sinusoidal endothelial cells of vascular regeneration can tolerate hypoxia or not, and if so, its specific mechanism. Briefly, mouse LSECs were cultured in hypoxia, and their proliferation ability was determined. Following this, the whole transcriptional group was sequenced to explore the specific molecular mechanism involved in LSEC proliferation. Finally, we explored the regulatory mechanism of LSEC proliferation at the transcriptome level by using high-throughput sequencing and bioinformatics methods. After identifying miRNAs and mRNAs that were differentially expressed (DE) in the LSECs, we analyzed and determined the biological processes and pathways of miRNAs involved in the regulation of mouse LSECs during hypoxia using bioinformatics. The main purpose of this study was to screen miRNAs and mRNAs involved in the proliferation of LSECs, which can provide more targets that can help further elucidate the role of LSECs in promoting LR after hepatectomy.

## 2. Materials and methods

### 2.1. Establishment of cell model and phenotype

Primary LSECs of mice were purchased from iCell Technologies Inc (Shanghai, China), Item No is MIC-iCell-d019 and the concentration of LSECs is more than 90%. Elastase and collagenase were used to digest mouse liver tissues to isolate LSECs from male C57BL/6 mice, and were then cultured in a primary endothelial cell culture medium at 37°C and 95% air and 5% CO_2_. The LSECs were inoculated in a 6-well culture plate, and the culture medium was changed every 24 h. After 2–3 days of primary culture, hypoxic culture (5% O_2_, 90% N_2_, and 5% CO_2_) was induced in the experimental group (group B) and normoxic culture (95% air and 5% CO_2_) in the control group (group A). After 24 h, total RNA was extracted from the cells of groups A and B for sequencing or for EdU staining to observe their proliferation activity. During hypoxic culture, serum-free or low-serum cultures were used.

### 2.2. 5-Ethynyl-2′-deoxyuridine (EdU) cell proliferation assay

LSECs were inoculated in 96-well plates (5 × 10^3^/well). After 24 h of simultaneous culture in Dulbecco’s modified Eagle’s medium (DMEM)/F12 containing fetal-free bovine serum, the cells were incubated in normoxic or hypoxic incubators for 24 h. Then, 100 μl EdU (50 µM; Guangzhou RiboBio Co., Ltd., Guangzhou, Guangzhou, China) was added to the wells and the Petri dish was incubated in a suitable chamber for 2 h according to the manufacturer’s instructions. After EdU staining, the cells were re-stained with 4′,6-diamidino-2-phenylindole (DAPI). The EdU-positive cells were randomly imaged by fluorescence microscopy(Olympus, Tokyo, Japan), and EdU-positive cells in five visual fields were counted randomly. The ratio of positive cells was calculated.

### 2.3. RNA isolation, library preparation, and sequencing

After total RNA extraction, the small RNA sequencing library was prepared using the TruSeq Small RNA Sample Prep Kit (Illumina, San Diego, CA, USA) kit. After the preparation of the library was completed, the constructed library was sequenced using Illumina Hiseq2000/2500, and the sequencing read length was single-ended 1 × 50 bp.

Raw data generated by sequencing was preprocessed. After quality control of the original data, the 3′ junction was removed by clean reads, and the length was screened to retain the sequence of base length of 18–26 nt. The remaining sequences were then compared with various RNA database sequences (excluding miRNA), such as mRNA database, RFam database, and Repbase database, filtered, and the final data obtained were considered the effective data, which was used for subsequent small RNA data analysis. The whole process was executed using the ACGT101-miR software (LC Sciences, Houston, TX, USA) Fold change (FC) > |1.2|, p < 0.05, was used as the cutoff criterion.

### 2.4. Statistical analysis of mRNA sequencing

Using group B as the experimental group and group A as the control group, combined with sequencing expression, analysis of the differential mRNA was carried out (screening criteria: FC > | 1.2 |, p < 0.05); In this process, we use transcript assembly software StringTie to assemble and quantify reads, and use R-packet edgeR for difference statistics and visual drawing.

### 2.5. Statistical analysis of miRNA sequencing

The differentially expressed miRNAs were identified, and target gene prediction was performed using miRanda and TargetScan databases [18].

### 2.6. Functional interpretation of differentially expressed (DE) genes

Gene Ontology (GO) analysis and Kyoto Encyclopedia of Genes and Genomes (KEGG) analysis of the screened common differential genes revealed that the GO entries and KEGG pathways were significantly enriched in the whole gene background. GO enrichment analysis can help roughly understand the molecular function (MF), cellular components (CC), and biological processes (BP) of differential genes. KEGG enrichment analysis can be used to infer the pathways through which the differential genes may play a role. In this study, the hypergeometric distribution method was used to enrich and analyze DE genes. In this method, the enrichment degree of a group of genes was evaluated by calculating the p-value.

(1) $$p=1-\overset{m-1}{\underset{i=0}{\sum }}\frac{\left(\begin{array}{c} M\\ i \end{array}\right)\left(\begin{array}{c} N-M\\ n-i \end{array}\right)}{\left(\begin{array}{c} N\\ n \end{array}\right)}$$

where, N is the number of genes with GO or KEGG annotation information for all genes, n is the number of differential genes in N, M is the number of genes annotated to a GO entry or KEGG pathway in all genes, and m is the number of differential genes annotated to a GO entry or KEGG pathway. Then, the p value was corrected by the false detection rate (FDR). p ≤ 0.05 of the corresponding GO entry or KEGG pathway was regarded as significant enrichment.

### 2.7. Construction of miRNA–mRNA regulatory network

When constructing the regulatory network of differential miRNA and target genes, the relationship strength of miRNA in the network, that is, the network eigenvalue of miRNA, is calculated according to the position function of miRNA in the network. The highest eigenvalue miRNA is in the pivotal position of the network, and this miRNA has the strongest regulatory ability and important regulatory value for the network structure and sample traits. At the same time, the key target genes regulated by miRNAs can also be obtained from the network.

### 2.8. Quantitative real-time PCR verification of mRNA and miRNA high-throughput data

Quantitative real-time PCR was used to detect miRNA and mRNA expression levels. After treatment, total RNA was extracted using TRIzol (Invitrogen, USA) following the manufacturer’s instructions. SuperScript III Reverse Transcriptase (Invitrogen, China) was used to synthesize cDNA was amplified using the SYBR Green qPCR Mix (Invitrogen). The Light Cycler Real-Time PCR System (Roche 480, Switzerland) was used to detect potential target miRNAs and mRNAs. The primer sequences were designed using Primer 5.0(Premier,Canada), and are shown in Table 1. *β-actin* and *U6* were used as the internal reference genes. The PCR conditions were as follows: 95°C for 3 min, followed by 40 cycles at 95°C for 15 s and 60°C for 30 s. The results are expressed as the fold difference relative to the level of *β-actin* or *U6* using the 2^−ΔΔCT^ method.

**Table 1.** Primers used for qRT-PCR

Gene		Sequence (5^,^ →3^,^)	product length
Klf4	Forward Primer	GCCAAAGAGGGGAAGAAGGT	135bp
	Reverse Primer	GGTTTCTCGCCTGTGTGAGT	
Smad2	Forward Primer	ATCTTGCCATTCACTCCGCC	96bp
	Reverse Primer	TCCATTCTGCTCTCCACCAC	
Thbs1	Forward Primer	CCAAGACCTACTGGACGCTG	108bp
	Reverse Primer	TCTTTCCGTTCCACAGCCAG	
Id4	Forward Primer	AGTCAGCAAAGTGGAGATCCTG	87bp
	Reverse Primer	CTCAGCAAAGCAGGGTGAGTC	
Ddit4	Forward Primer	TGTGTGTGGAGCAAGGCAAG	89bp
	Reverse Primer	CACCAGGGTCAACTGAAAGGT	
Lifr	Forward Primer	GATGTGTACGGAACGGTGGT	129bp
	Reverse Primer	GCCCACCAGTCCAGTTATCC	
Tnfaip3	Forward Primer	TCACTCCACACTCTTGCCAC	93bp
	Reverse Primer	TTGTCCCTGCTCTGTCTCCT	
Wnt7a	Forward Primer	GGCCACCTCTTTCTCAGCC	83bp
	Reverse Primer	ATGATGCTCGCACCCAGAG	
Csf1	Forward Primer	TGCCAAGGAGGTGTCAGAAC	240bp
	Reverse Primer	CTCTCGGTGGCGTTAGCATT	
Gsk3b	Forward Primer	TCCACATGCTCGGATTCAGG	101bp
	Reverse Primer	AAGCGGCGTTATTGGTCTGT	
Tnfrsf1b	Forward Primer	GCAGCTCTGACCACAGTTCT	112bp
	Reverse Primer	TCCTGAGAGAAGGGGACCTG	
Csf2	Forward Primer	ACTTTTCCTGGGCATTGTGG	85bp
	Reverse Primer	CTCTACATGCTTCCAAGGCCG	
β-actin	Forward Primer	GATATCGCTGCGCTGGTCG	132bp
	Reverse Primer	CATTCCCACCATCACACCCT	
mmu-miR-199b-3p	Forward Primer	ACAGTAGTCTGCACATTGGTTA	
mmu-miR-152-3p	Forward Primer	TCAGTGCATGACAGAACTTGG	
mmu-miR-543-3p	Forward Primer	AAACATTCGCGGTGCACTTCTT	
mmu-miR-130b-5p	Forward Primer	TCCCTGAGACCCTTTAACCTGTGA	
mmu-miR-125a-5p	Forward Primer	TCCCTGAGACCCTTTAACCTGTGA	
mmu-miR-126a-5p	Forward Primer	CATTATTACTTTTGGTACGCG	
U6	Forward Primer	GGAACGATACAGAGAAGATTAGC	103bp
	Reverse Primer	TGGAACGCTTCACGAATTTGCG	

### 2.9. Statistical analyses

Statistical analyses were performed using GraphPad Prism 8.1 (GraphPad Software, Inc., La Jolla, CA). The data are presented as the mean ± standard deviation (SD). Real-time PCR data were analyzed with Student’s t-test and a p < 0.05 indicates a statistically significant difference.

## 3. Results

Based on previous reports on the initial stages of LR, in this study, the process of mouse hepatocyte regeneration was carried out in a hypoxic environment. We also analyzed whether the sinusoidal endothelial cells of vascular regeneration can tolerate hypoxia or not, and if so, its specific mechanism. Therefore, in this study, firstly, the primary LSECs of mice were cultured in hypoxia and found that its proliferation ability was enhanced. In order to explore its specific mechanism, we used high-throughput sequencing and bioinformatics methods to explore the regulation mechanism of LSECs proliferation at the transcriptome level during hypoxia. The main purpose of this study was to screen miRNAs and mRNAs involved in the proliferation of LSECs, which can provide more targets that can help further elucidate the role of LSECs in promoting LR after hepatectomy. Next, the results of the study will be presented one by one.

### 3.1. Proliferation of LSECs was enhanced in hypoxic culture

**Figure 1.**
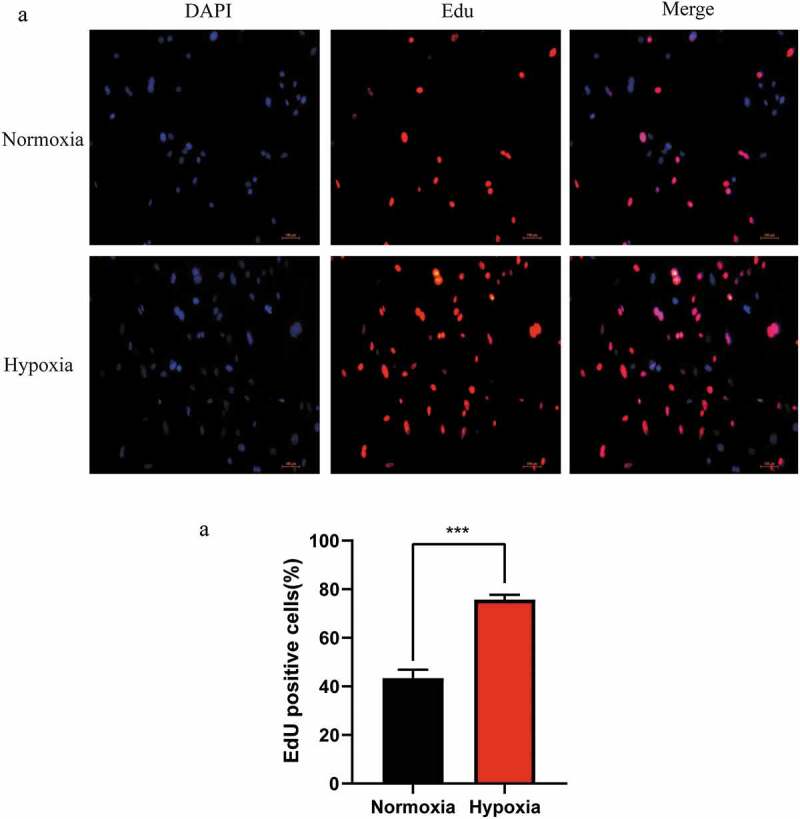
Changes in the proliferation of mouse liver sinusoidal endothelial cells (LSECs) cultured under hypoxic conditions. (a) Representative images of EdU staining assay of mouse LSECs cultured under hypoxia or normoxia for 24 h. (b) The average number of positive cells in Edu staining after LESCs were cultured in hypoxia or normoxia for 24 h. Data are shown as the mean ± SEM. n = 3 per group,***p < 0.001 vs control group (normoxia) by Student’s t-test

To investigate the effect of the hypoxic microenvironment in promoting liver regeneration during the initial stages was related to LSECs, the proliferation of LSECs in hypoxia was determined using the EdU staining method. It was found that the proliferation ability of LSECs was stronger than that of the control group (normoxic group) after hypoxic culture for 24 h (Figure 1). Therefore, it can be inferred that hypoxia promotes LR by regulating LSECs during the initial stage of LR.

### 3.2. Determination of differentially expressed (DE) mRNAs and differentially expressed miRNAs (DE) after hypoxia culture

The initial stage of LR occurs in a hypoxic microenvironment, and we confirmed that the proliferation of LSECs was enhanced during hypoxia. In order to explore the specific molecular mechanisms involved, we carried out high-throughput sequencing of the hypoxic-cultured LSECs (group B) and normoxic-cultured LSECs (group A) with three samples in each group, and the DE mRNA and DE miRNAs were obtained. Compared with the control group, we detected a total of 1837 genes with significant differential expression, including 1056 upregulated and 781 downregulated genes, while 17 miRNAs showed significant differences, including 7 upregulated and 10 downregulated miRNAs (Tables S1 and S2).

We then used miRanda and TargetScan databases to predict the target genes of the screened DE miRNAs. The result of the target genes was then intersected with the screened DE mRNAs, and the screened mRNAs were then paired with the miRNAs to obtain the negative correlation pair of miRNA–mRNA. (Table S3).

### 3.3. Biological function prediction of differentially expressed (DE) mRNAs

**Figure 2.**
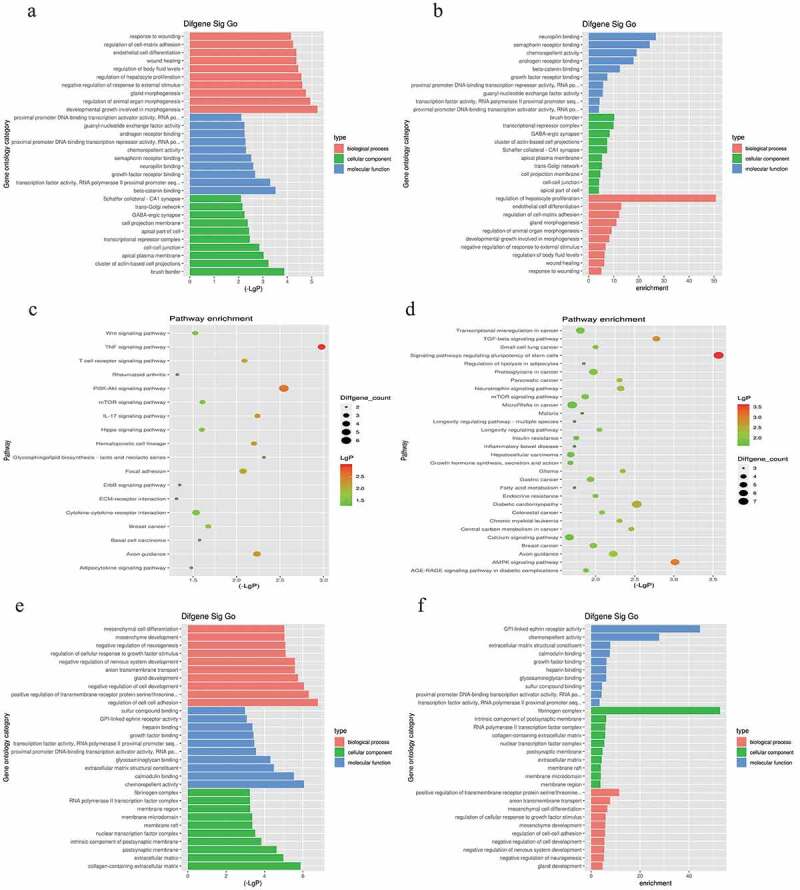
Gene Ontology (GO) and Kyoto Encyclopedia of Genes and Genomes (KEGG) enrichment analysis based on miRNA-targeted genes. (a), (b), and (c) illustrate significant GO-biological processes (BP) enrichment analysis of MF (molecular functions), CC (cellular components), and pathways, respectively, targeted by downregulated genes. Similarly, (d), (e), and (f) show the same content in upregulated genes

We analyzed the potential biological functions of DE mRNAs based on the GO enrichment regions (including BP, CC, and MF)(Tables S4 and S5). In addition, KEGG enrichment was performed to concentrate on the expression of the signaling pathways of mRNAs that were fully involved (Tables S4 and S5). Functional analysis of DE mRNAs indicated that the most significantly enriched BP were regulation of hepatocyte proliferation, endothelial cell differentiation, regulation of cell-cell adhesion, and regulation of cellular response to growth factor stimulus (Figure 2). Hepatocyte proliferation regulation and endothelial cell differentiation regulation were ranked among the top ten enriched BP. Pathways including the TNF signaling pathway, PI3K-Akt signaling pathway, and signaling pathways regulating the pluripotency of stem cells may be closely enriched in LSECs under hypoxic conditions.

**Figure 3.**
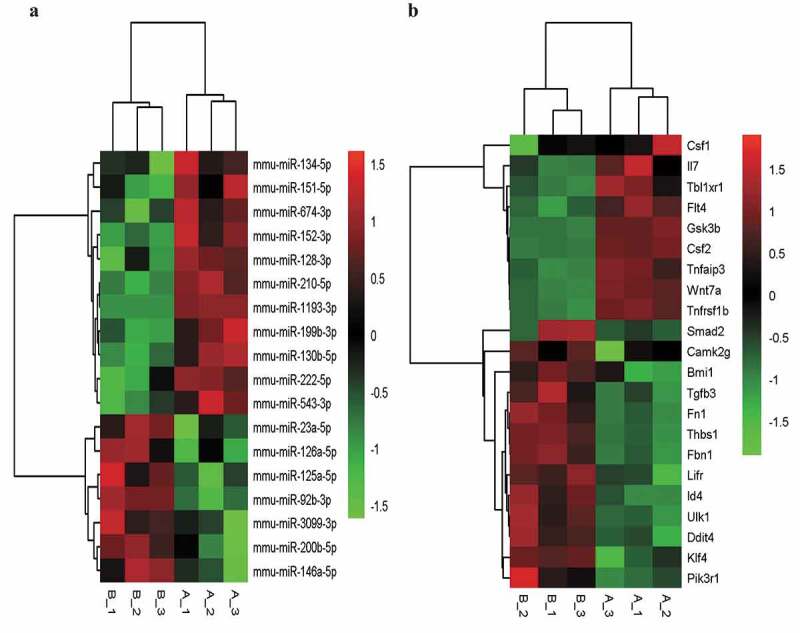
The fine cluster map of miRNA and its predicted target genes were significantly altered in mouse liver sinusoidal endothelial cells (LSECs) cultured in hypoxia and normoxia (control group) for 24 h. (a) The expression profile of miRNA in mouse LSECs after 24 h of hypoxia and normoxia (control group). A1, A2, A3 served as control groups. (b) The target genes predicted by differentially expressed (DE) miRNA were enriched by GO-BP and KEGG, and the target intersecting genes related to LSECs and hepatocyte proliferation were intersected by the list of genes enriched in pathway and function. Combined with their initial expression values, the fine cluster map of mRNA was obtained. A total of 22 meaningful DE target genes were obtained, including 13 upregulated genes and 9 downregulated genes

We then selected the BP and signal pathways related to LSEC proliferation that were significantly enriched in GO-BP and KEGG. The target intersecting genes were obtained by intersecting the list of genes in the pathways and concerned functions, and the fine clustering map was created according to the initial expression value (fragments per kilobase of exon per million mapped fragments, FPKM) of the genes (Figure 3).

### 3.4. Predictive results of miRNA–mRNA interactions

**Figure 4.**
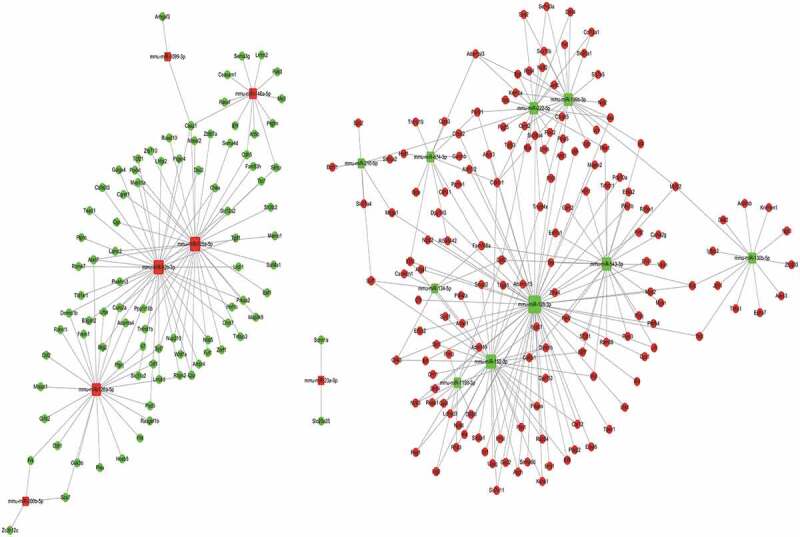
An interaction network of the miRNAs and their targets in liver sinusoidal endothelial cells (LSECs) cultured under hypoxia for 24 h

Based on the sequencing results, we then attributed relationship between 17 miRNAs with significant differential expression, their targeting genes, and the negative correlation results of differential genes to establish the miRNA–gene action network (Figure 4). Our constructed network produced 309 relationships among miRNAs and mRNAs using TargetScan and miRanda. Through the miRNA–mRNA interaction network, we obtained the differential changes in core miRNA and genes in LSECs after hypoxia culture, which may explain the molecular mechanism involved in the enhancement of LSEC proliferation ability in hypoxia, and clarify the molecular mechanism of liver regeneration from the point of view of LSECs.

### 3.5. Validation of differentially expressed (DE) miRNA and mRNA

**Figure 5.**
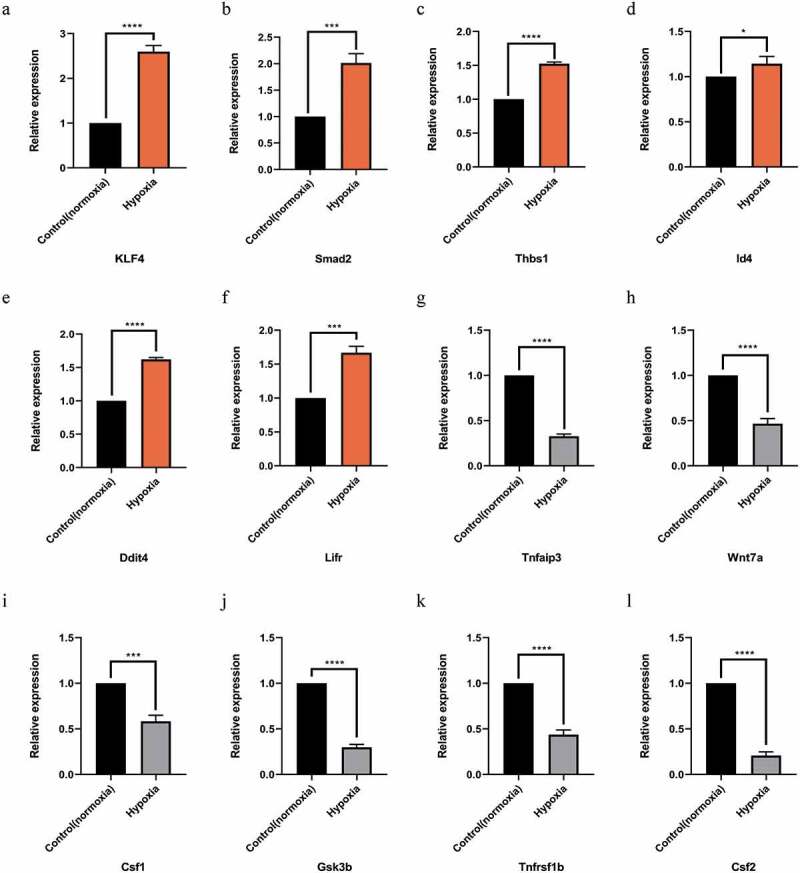
Quantitative real-time PCR (qPCR) validation of the differentially expressed mRNAs (TOP 6) identified by transcriptomic analysis. (a),(b),(c),(d),(e),(f) show up-regulated mRNAs; (g),(h),(i),(j),(k),(l) show down-regulated mRNAs .Data are shown as the mean ± SEM,n = 3 per group,*p < 0.05, ***p < 0.001,****p < 0.0001 vs control group (normoxia), by Student’s t-test

**Figure 6.**
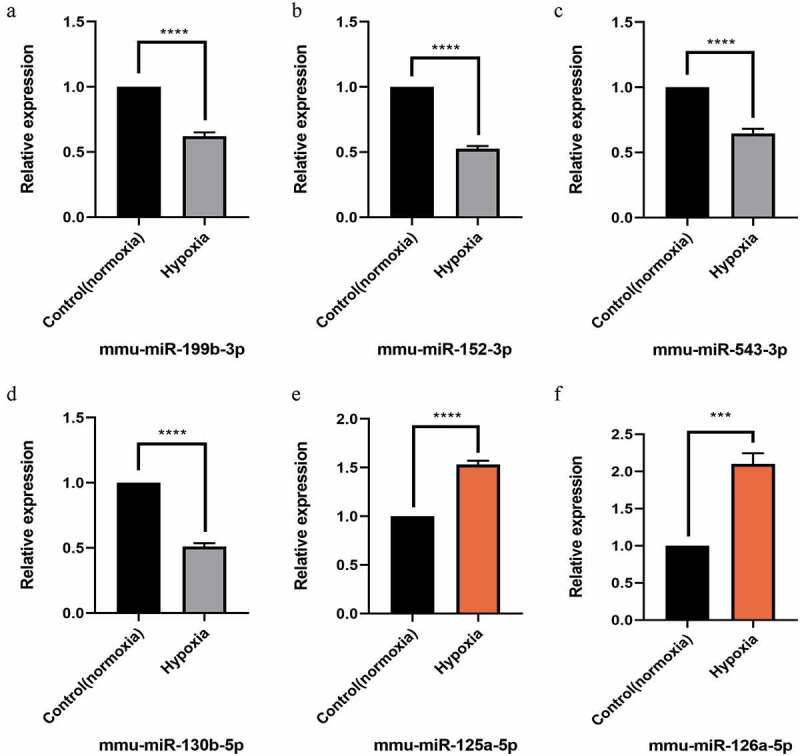
Quantitative real-time PCR (qPCR) validation of the differentially expressed miRNAs (TOP 6) identified by transcriptomic analysis.(a),(b),(c),(d) show down-regulated miRNAs;(e),(f) show up-regulated miRNAs. Data are shown as the mean ± SEM, n = 3 per group, ***p < 0.001,****p < 0.0001 vs control group (normoxia), by Student’s t-test

After the intersection of miRNA-predicted target genes and significantly differentially expressed mRNAs, the genes related to LSEC proliferation in GO-BP and KEGG were identified and then combined with their initial expression values. The top six up-and down-regulated genes were further selected for RT-PCR verification, and their corresponding miRNA expression according to the miRNA-mRNA interaction network was also verified (Figure 5 and Figure 6).

## 4. Discussion

Previous studies have revealed that LSECs play a vital role in LR [9,10,19,20]. Furthermore, studies have also confirmed that during the initial stage of LR, hepatocytes and LSECs initiate regeneration in a hypoxic environment [11,21]. However, how LSECs participate in liver regeneration in hypoxic environments and the specific molecular mechanism is unknown. Hence, we aimed to study of the effect of LSECs on LR, especially the importance of the hypoxic microenvironment in promoting LR. We found that hypoxia promoted the proliferation of LSECs. Moreover, an increasing number of recent studies have found that miRNA-mediated regulation plays an important role in controlling the proliferation of hepatocytes and LSECs during LR [22–25]. However, the specific mechanism is unknown. Hypoxia can regulate the effects of LSECs on promoting LR through miRNAs. We explored the regulatory mechanism of miRNA on hepatic sinusoidal endothelial cell proliferation and LR by hypoxia treatment of primary hepatic sinusoidal endothelial cells, followed by full transcriptional sequencing to observe the changes in miRNA and mRNA.

In this study, high-throughput sequencing was conducted to analyze the expression changes in miRNAs and mRNAs in mouse LSECs cultured under hypoxia. Based on the RNA sequence data, 17 DE miRNA and 1837 DE mRNA were identified in LSECs after 24 h of hypoxic culture compared to the control group. In this study, from the perspective of LSECs, we simulated the hypoxic state of the hepatic sinusoidal environment in the early stage of LR, and then detected the differential changes of miRNA and mRNA in LSECs, and analyzed their effects on the biological process of LSECs, especially on proliferation, in order to regulate LR. None of the DE miRNAs have been previously studied in the proliferation of LSECs nor in LR. In order to study the role of miRNAs in the proliferation of LSECs after hypoxic culture, we predicted the target genes using miRanda and TargetScan database [26], and then selected the target genes by intersection with differential mRNAs. GO enrichment analysis of the target mRNAs was then performed. The results indicated that a large number of significant GO terms were related to the regulation of hepatocyte proliferation, cell adhesion, cellular response to growth factor stimulus, and regulation of cell growth, which are some biological processes related to proliferation or involved in regulating the proliferation of liver cells. Furthermore, it is suggested that the changes in the expression of miRNA and mRNA in hypoxia-induced LSECs may be involved in the regulation of LSECs and hepatocyte proliferation. It has been suggested that LSECs are involved in the regulation of LR. We found that the upregulated genes were enriched in 50 signal pathways, while the downregulated genes were enriched in 18 signaling pathways. The pathways involved in the TNF, PI3K-Akt, mTOR, HIF-1, AMPK, Hippo, Wnt, and IL-17 signaling pathways and cytokine-cytokine receptor interaction may be closely enriched in LSECs cultured in hypoxia during the early stage of LR .

In order to further clarify the role of miRNAs in hypoxic cultures of LSECs, we established a miRNA–gene network based on the attribute relationship between 18 significantly differentially expressed miRNAs and their targeted genes and negative correlation results of differential genes. In the miRNA–mRNA interaction network, six core miRNAs (miR-126a-5p, miR-92b-3p, miR-125a-5p, miR-152-3p, miR-128-3p, and miR-543-3p) were identified according to the literature and the regulatory relationship between miRNAs and mRNAs. These core miRNAs may be involved in regulating the proliferation of LSECs after hypoxic culture and even participate in the regulation of hepatocyte proliferation.

Finally, we selected the functions and signal pathways related to this study, such as the signaling pathways related to proliferation, and then intersected the concerned pathways and functional gene lists to obtain the target intersection gene. Combined with its initial expression value, we determined that the top six upregulated genes were *Klf4, Smad2, Thbs1, Id4, Ddit4*, and *Lifr*. Similarly, the top six downregulated genes were *Tnfaip3, Wnt7a, Csf1, Gsk3b, Tnfrsf1b*, and *Csf2*. Furthermore, significantly different and significant top six miRNAs, of which the downregulated miRNAs were mmu-miR-199b-3p, mmu-miR-152-3p, mmu-miR-543-3p, and mmu-miR-130b-5p, and upregulated mmu-miR-125a-5p and mmu-miR-126a-5p were also identified.

Some studies have shown that miRNAs are involved in regulating the whole process of LR, and studies have found that miR-199a-5p may help to improve hepatocyte production and LR *in vivo* [25,27,28]. miR-125b-5p regulates hepatocyte proliferation at the termination of LR [29]. In addition, miRNAs are also involved in the regulation of various pathophysiological processes of hepatic sinusoidal endothelial cells; for example, miR-511-3p is involved in the regulation of LSEC injury in hepatic sinusoid obstruction syndrome [30–32]. However, whether miRNAs regulate the effects of LSECs on promoting LR is yet to be reported.

Finally, we verified the top six selected up- and down-regulated genes and the corresponding miRNAs by RT-PCR, and found that the PCR results were consistent with the results of Qualcomm sequencing, indicating that the sequencing results are accurate and reliable.

In this study, based on hypoxia promoting the proliferation of LSECs, it was concluded that mRNA and miRNA, which may promote the proliferation of LSECs and hepatocytes, respectively, provide a new target for further exploring the effect of LSECs on promoting LR. Nevertheless, this study still has some limitations. This study used mouse primary LSEC cells cultured in hypoxia *in vitro*, which cannot completely and objectively reflect the complex microenvironment in animals in the early stage of LR. We strive to be able to use animal models for experiments in our future studies to obtain more objective and accurate data.

## 5. Conclusion

This study proposed that a specific miRNA–mRNA network is associated with LSEC proliferation in hypoxia, which will possibly assist in elucidating the potential mechanisms involved in hypoxia-promoting liver regeneration. First, DE miRNAs and DE mRNAs were analyzed using high-throughput sequencing technology, and then the miRNA–mRNA regulatory network was constructed according to the regulation mechanism of miRNA. Using high-throughput sequencing and the bioinformatics method, we constructed a miRNA–mRNA regulatory network and identified 6 key miRNAs and 12 significant differences in the expression of mRNAs, which may play an critical role in LSECs in the process of promoting liver regeneration. However, this study has some limitations that warrant further detailed studies in the future.

## Supplementary Material

Supplemental Material

## Data Availability

The data used to support the findings of this study are available from the corresponding author upon request.

## Highlights:

1. The proliferation activity of LSECs was enhanced in hypoxic culture.

2. Differentially expressed miRNA and mRNA regulate LSEC proliferation.

3. Hypoxia induces a specific miRNA–mRNA network during LSEC proliferation.

## Abbreviations

LSECs, liver sinusoidal endothelial cells; DE, differentially expressed; LR, liver regeneration; KEGG, Kyoto Encyclopedia of Genes and Genomes; GO, Gene ontology; BP, Biological Process; mRNA, messenger RNA; miRNA, micro RNA, EdU, 5-Ethynyl-2′-deoxyuridine; qRT-PCR, quantitative real-time polymerase chain reaction; CC, cellular components; MF, molecular functions; FDR, false discovery rate;FC, fold change.
